# Addiction to the nicotine gum in never smokers

**DOI:** 10.1186/1471-2458-7-159

**Published:** 2007-07-17

**Authors:** Jean-François Etter

**Affiliations:** 1 Institute of social and preventive medicine, University of Geneva, 1 rue Michel-Servet, CH-1211 Geneva 4, Switzerland

## Abstract

**Background:**

Addiction to nicotine gum has never been described in never smokers or in never users of tobacco.

**Methods:**

Internet questionnaire in 2004–2006 in a self-selected sample of 434 daily users of nicotine gum. To assess dependence on nicotine gum, we used modified versions of the Nicotine Dependence Syndrome Scale (NDSS), the Fagerström Test for Nicotine Dependence and the Cigarette Dependence Scale.

**Results:**

Five never smokers used the nicotine gum daily. They had been using the nicotine gum for longer than the 429 ever smokers (median = 6 years vs 0.8 years, p = 0.004), and they had higher NDSS-gum Tolerance scores (median = 0.73 vs = -1.0, p = 0.03), a difference of 1.5 standard deviation units. Two never smokers had never used smokeless tobacco, both answered "extremely true" to: "I use nicotine gums because I am addicted to them", both "fully agreed" with: "after a few hours without chewing a nicotine gum, I feel an irresistible urge to chew one" and: "I am a prisoner of nicotine gum".

**Conclusion:**

This is to our knowledge the first report of addiction to nicotine gum in never users of tobacco. However, this phenomenon is rare, and although the long-term effect of nicotine gum is unknown, this product is significantly less harmful than tobacco.

## Background

Nicotine replacement therapy (NRT) is effective to treat tobacco dependence, and about one third of smokers ever used NRT products [1-3]. In many countries, NRT products can be obtained on general sales, in drugstores or on the internet, without a medical prescription and without counselling [3]. NRT products are therefore easily accessible to nonusers of tobacco. Since nicotine has the potential to be addictive, there is a possibility that non-users of tobacco may become addicted to NRT products. There is to our knowledge, however, no published case of addiction to NRT products in never smokers or in never users of tobacco. Among former smokers who used the nicotine gum to quit smoking, the prevalence of dependence on the gum is very low, about one percent [4-6]. The proportion of never-smokers and never-users of tobacco among regular nicotine gum users is unknown and, contrary to what many people believe, this proportion may not necessarily be zero. The aim of this study was to assess whether we could identify never-smokers and never-users of tobacco who used the nicotine gum or were addicted to it.

## Methods

We posted a questionnaire on nicotine gum use on the internet between September 2004 and March 2006, in English, on the smoking cessation website [7,8]. The questionnaire was listed second on Google.co.uk when typing "nicotine gums" (tested November 8, 2006).

A link to the questionnaire was posted on other smoking cessation websites. The questionnaire is available at [9]. This study was not submitted to an ethics committee, but participants were informed that their answers were stored on file and had the option to refuse storage of their data.

Questions included: Have you ever used nicotine replacement products (i.e. nicotine gums, patches, tablets or inhalers)? (Yes, no); Do you currently use nicotine replacement products? (Every day, occasionally, used in the past, never used); Currently, on how many days per week do you use nicotine chewing gums?; How long did your current episode of nicotine gum use last?; Currently, how many nicotine chewing gums do you use per day? Questions also covered intention to stop using the gum, serious attempts to stop using nicotine gum in the past year, duration of the last attempt to stop using the gum and urges to use nicotine gum during the last quit attempt. We defined never smokers as people who answered "No" to: "Have you ever smoked 100 cigarettes or more in your lifetime?" and: "No, I never was a smoker" to: "Do you currently smoke tobacco?". Use of smokeless tobacco was assessed by email, in never smokers who reported using nicotine gum daily in the online questionnaire.

Because we know of no published, validated questionnaire measuring dependence on the nicotine gum, we modified the Fagerström Test for Nicotine Dependence (FTND), the Nicotine Dependence Syndrome Scale (NDSS) and the Cigarette Dependence Scale (CDS) to assess gum dependence [10-13]. FTND and CDS are unidimensional, whereas NDSS covers 5 dimensions of dependence: Drive, Priority, Tolerance, Continuity and Stereotypy [11]. In all items, we replaced the words "cigarette" by "nicotine gum", and "smoking" by "using" or "chewing nicotine gum". We computed NDSS standardized scores as recommended [11]. We dropped the NDSS item: " Even if travelling a long distance, I'd rather not travel by airplane because Iwouldn't be allowed to smoke", because it cannot easily be modified for nicotine gum use. We modified the NDSS item "I tend to avoid restaurants that don't allow smoking, even if I would otherwise enjoy the food" to: "I tend to avoid places where I cannot chew nicotine gum, even if I would otherwise enjoy the company". We used Mann-Whitney U-tests to compare medians and chi-square tests to compare proportions.

## Results

There were 848 participants in the online survey, including 436 daily users of nicotine gum, 68 occasional (non-daily) users and 344 non-users. Among the 434 daily gum users who indicated their smoking status, there were 349 former smokers (80%), 80 current smokers (18%) and 5 never smokers (1.2%). The median age of these 5 never smokers was 44 years (range 38 to 59 years), they comprised 3 women and one man (one missing answer), and 3 people who lived in the USA and one in the UK (one missing answer).

Compared with the 429 ever smokers who used the gums daily, the 5 never smokers tended to have higher nicotine gum dependence scores for all FTND-gum, CDS-gum and NDSS-gum scales, but statistically significant differences were observed only for the NDSSgum Tolerance score (never smokers: median = 0.73, ever smokers: median = -1.0, Mann-Whitney U-test: p = 0.03), which represents a difference of 1.5 standard deviation units. Three of the 4 never smokers who answered this NDSS question, but only 14% of ever smokers answered "extremely true" to: "compared to when I first started using nicotine gum, I can use many, many more nicotine gum now before I start to feel nauseated or ill" (chi-square = 13.2, p = 0.01). In addition, the duration of the current episode of nicotine gum use was longer in the 5 never smokers than in the 429 ever smokers (median = 6 years vs 0.8 years, Mann-Whitney U-test: p = 0.004).

We obtained more information by e-mail from 4 of the 5 never smokers who used the nicotine gum daily. Three of these 4 people confirmed that they had never smoked, but one woman said she had smoked a few cigarettes occasionally. Three said they had never used smokeless tobacco, but one man said he had used small amounts of tobacco snuff as a child, decades before he started using the nicotine gum. They authorized us to report their comments.

E., female, 38, USA, was using the nicotine gum for 2 years, used 5 gums/day

"I've never smoked. My sister had some nicotine gum when she was trying to quit smoking, and I happened to try a piece. It seemed to wake me up and give me an energy boost, which I liked. I don't drink coffee, so I use the gum to wake me up when I'm feeling drowsy."

S., female, 39, USA, was using the nicotine gum for 10 years, used 15 gums/day

"I have never smoked cigarettes. I started using nicotine gum by chance, when a medical student offered them to me to try. I still use them because I am addicted to nicotine, and can not seem to quit using them even if have tried."

In e-mail messages, these two participants (E. and S.) answered that they had never used smokeless tobacco, had never abused or been dependent on other substances and had never been diagnosed with a psychiatric illness. In the online questionnaire, these 2 never-users of tobacco reported addiction levels to the gum of 95 and 98 on a scale of 0–100, FTND-gum ratings of 2 and 5, CDS-gum ratings of 44 and 56 and NDSS-gum ratings of -0.9 and 0.42.

Both answered "extremely true" to: "I use nicotine gums because I am addicted to them" and: "I use nicotine gum because I a cannot stop using them" and: "I use nicotine gum to deal with stress". Both reported that stopping using all NRT products would be "very difficult". Both "fully agreed" with: "after a few hours without chewing a nicotine gum, I feel an irresistible urge to chew one", "I am a prisoner of nicotine gum" and: "after chewing a nicotine gum, I am able to concentrate better". Both "seriously considered" stopping NRT in the next 6 months. One had made a serious attempt to stop using the gums during the previous year, during which she felt a "very strong" urge to use nicotine gum. This failed quit attempt lasted 30 days.

In the online survey, three additional participants reported being never smokers. Two of them could be further contacted by e-mail:

G., female, 59, USA, was using the nicotine gum for 10 years, used 10 gums/day

"During the time [my husband] was a smoker, I was a "social smoker". I would smoke at parties [his cigarettes] and occasionally at home after dinner or with a drink. I never bought or carried cigarettes with me, I only smoked his or on occasion my co-worker's. [...] About 15 years ago, my husband [...] started chewing prescription Nicorette, and I chewed it occasionally as well. When it became available over the counter, we both just continued to chew it, and I became more and more addicted to it. I started with one or two pieces occasionally, then one or two a day, and finally up to a dozen pieces a day, and continued at the rate for several years, maybe 4–5 years [...]. It is really awful to be addicted to a substance, and I reached a point where my throat hurt, my teeth hurt, I had a headache most days, [...] but I still kept chewing. And it is so expensive – I was spending over a $100 a month on it."

In an e-mail message, this participant (G.) said she had never used smokeless tobacco and had never abused or been addicted to other substances. She had been diagnosed with depression after a severe traumatic stress and took antidepressants (paroxetine) for several years after that. She said: "I think the depression was a natural reaction to [the traumatic stress]. Nicotine was part of my self-medication duringall this".

A., male, 49, UK, was using the nicotine gum for 6 years, used 20 gums/day

This participant reported that he had occasionally used small quantities of tobacco snuff as a child, between the age of 11 and 14, but not any more since then. He also said he had suffered from depression and had been prescribed fluoxetine by his family doctor. He reported:

"I have never smoked any tobacco product or even marijuana in my life. I have experimented with LSD, psilocybin, amphetamines but not currently using any of these. Several years ago, purely out of interest I bought a pack of nicotine gum to see what the effect of nicotine would be. I remember feeling quite ill [...]. It was about a year or so later [...], I remembered my pack of nicotine gums and started to use them maybe two or three times a day. I didn't expect that I would ever become addicted [...]. Within a short period of time I was using 15 pieces per day. Just as people say about smoking, I genuinely enjoyed using the gum [...]. On several occasions I stopped using it 'cold turkey'. The symptoms were mainly light headedness and difficulty in concentration, most of the time I just bought more gums – like a smoker [...]. Most of my life I have suffered from depression [...]. I hadn't considered until then that nicotine could have such a stabilising effect. I would very much like to quit. The gum is very expensive [...]. Nicotine gum is a form of self-medication [...]. I would argue that my state of mind has become more calm and regulated since using nicotine gums and reverts to previous, unsatisfactory state when I stop using nicotine gums. [...] I started using the illicit drugs above shortly before using nicotine gums on a regular basis."

These two participants (G. and A.) reported levels of addiction to the gums of 80 and 95 on a 0–100 scale, FTND-gum ratings of 5 and 6, CDS-gum ratings of 50 and 55 and NDSS-gum ratings of -0.21 and 0.67. Both answered "extremely true" to: "I use nicotine gums because I am addicted to them". Both had made a serious attempt to stop using NRT in the previous year, but only one reported more details about that quit attempt, which lasted 60 days and was accompanied with "very strong" urges to use nicotine gum. Both reported usually chewing their first gum of the day 10 minutes after waking up, both "fully agreed" with: "after a few hours without chewing a nicotine gum, I feel an irresistible urge to chew one", "the idea of not having any nicotine gum causes me stress", "sometimes I drop everything to go out and buy nicotine gums", "chewing a nicotine gum calms me down when I am stressed", and: "after chewing a nicotine gum, I am able to concentrate better".

## Discussion

In an online study of nicotine gum users, we identified 5 never smokers who used the nicotine gum daily. There was reasonable evidence that most of these 5 never smokers were addicted to the nicotine gum. Of particular interest are the two cases of never users of tobacco (including smokeless tobacco) who reported being addicted to the nicotine gum. This is to our knowledge the first report of such cases.

Surprisingly, these 5 never smokers had been using the nicotine gum for many more years than ever smokers, and they tended to be more dependent on the gum. For cigarette dependence, FTND scores of 5 or more reflect a strong dependence [14,15]. Four of the 5 never smokers had FTND-gum scores of 5 or more, which suggests that these people were strongly dependent on the gum. In a previous study, the average CDS score in daily smokers was 47, and daily smokers who had CDS scores >=50 smoked on average 25 cigarettes per day and smoked their first cigarette within 10 minutes of waking up [13]. Three of the 5 never smokers in the present study had CDS-gum scores at or above 50, which suggest that they were strongly dependent on the nicotine gum. Never smokers had substantially higher NDSSgum Tolerance scores than ever smokers, which may reflect that ever smokers were already tolerant to nicotine before they started using the gum.

Never-smokers reported classical criteria of dependence, such as a compulsion to use the gum, use in higher dosage and for a longer duration than initially intended, withdrawal symptoms, unsuccessful attempts to stop and gum use despite its high cost [16,17]. Their open-ended answers also reflected classical criteria of dependence, such as failed quit attempts followed by urges to use the gum, relief of withdrawal symptoms (e.g. difficulty concentrating) by gum use, and withdrawal symptoms (e.g. urges to use the gum after a few hours).

People who were addicted to the nicotine gum could easily find our questionnaire, because it was listed on top of the list in Google. In spite of this effective enrolment strategy, we identified only two never-users of tobacco among daily gum users, which suggests that NRT use in never-users of tobacco is a rare phenomenon. Similarly, a previous survey in people who responded to a newspaper ad that read: "Are you addicted to nicotine gum?" could not enrol any never smoker [5]. Furthermore, there was no report of subsequent nicotine dependence in never smokers who were treated with nicotine for ulcerative colitis, aphtous ulcers and sleep-disordered breathing [18-21]. The short-tem effect of the nasal spray was also tested in never smokers, with no report of never smokers getting addicted to this fast delivery product [22-24]. In a previous survey, 0.3% of adolescent never smokers reported past daily use of NRT, but none was reported as being addicted to NRT [25]. However, some adolescents will endorse using any product when a list is presented to them, e.g. 0.4% said they used a fictitious nicotine "Nic-T" product [26]. In two surveys in the USA, 2.7% and 4.6% of school drug counsellors indicated that nicotine patches and gums were abused by adolescents, but these "NRT abusers" were mainly smokers who used NRT while smoking, and only 7% to 16% of these "NRT abusers" were never smokers [27]. The latter study did not report any case of NRT dependence in adolescent never smokers [27]. Similarly, studies in representative samples of the UK and Swedish general populations found no never-user of tobacco among users of NRT [28]. A review of post-marketing surveillance data in the USA found no report of primary dependence to the nicotine gum and patch,[29] and only 39 cases of dependence on the nicotine gum were reported per million prescriptions to smokers, in surveillance data [30]. Therefore, addiction to nicotine gum in never smokers is probably very rare. Furthermore, there may be few adverse consequences of being addicted to the nicotine gum, except for the financial cost and the inconvenience of permanent chewing. In particular, NRT products are safe even in patients with heart disease, [31,32] and there was no untoward effect of 5 years of nicotine gum use in the Lung Health Study [33]. Thus, long-term use of NRT is not known to be harmful.

Participants volunteered to take part in our study after finding a link to the questionnaire on Google. This enabled anyone who was looking for information on the nicotine gum to find the questionnaire easily. As a result, our sample consisted of a self-selected group of people who proactively searched the web for information on the nicotine gum, perhaps because they worried about their nicotine gum use. This sample is therefore not representative of all nicotine gum users. In particular, only 2% of USA households who purchased nicotine gums purchased them monthly for 6 months or more,[34] and only 6% of participants in a nicotine gum trial used the gum for 24 weeks or more [35] These figures contrast sharply with the median duration of 8 months of NRT use in ever smokers in our survey, which suggests that our method oversampled long-term users of NRT. It is therefore not possible to extrapolate from our data a population prevalence of never smokers among daily gum users. Our aim was however not to determine this prevalence, but to test whether we could identify any never-user of tobacco or any non-smoker among nicotine gum users. Our findings have important implications for the understanding of the effects of nicotine, since it was believed until now that nicotine addiction could be maintained, but not initiated by the nicotine gum [5].

## Conclusion

Addiction to the nicotine gum in never smokers is probably quite rare, but this study suggests that it can occur.

## Competing interests

The Institute of Social and Preventive Medicine of the University of Geneva received financial support from Pfizer and Novartis to develop, under the supervision of the author, online smoking cessation programs for users of nicotine replacement products. The author acted as a consultant for Pfizer.

## Funding

No external funding.

## Pre-publication history

The pre-publication history for this paper can be accessed here:


